# Gender-based differences in decision making, quality of life and survival among patients undergoing left ventricular assist device evaluation

**DOI:** 10.1371/journal.pone.0293121

**Published:** 2023-10-25

**Authors:** Joseph M. Kim, Muhammad H. Maqsood, Tracy Makuvire, Colleen K. McIlvennan, Jocelyn S. Thompson, Daniel D. Matlock, Larry A. Allen, Haider J. Warraich

**Affiliations:** 1 Division of Cardiovascular Medicine, Beth Israel Deaconess Medical Center, Boston, MA, United States of America; 2 Department of Internal Medicine, Lincoln Medical and Mental Health Center, New York, NY, United States of America; 3 Department of Medicine, Brigham and Women’s Hospital, Boston, MA, United States of America; 4 Department of Medicine, University of Colorado School of Medicine, Aurora, CO, United States of America; 5 Department of Medicine, Cardiology Section, VA Boston Healthcare System, Boston, MA, United States of America; PLOS ONE, UNITED KINGDOM

## Abstract

**Background:**

Women are less likely to receive left ventricular assist devices and are more likely to experience poor outcomes. However, how gender impacts LVAD decision-making regarding LVAD implantation and the effects of that decision remains unknown.

**Methods:**

We performed a sub-group analysis from the stepped-wedge DECIDE-LVAD trial, which tested a decision-support intervention for patients considering LVAD therapy.

**Results:**

Excluding 9 patients who withdrew from assessments, of the 239 patients analyzed, 203 (85%) were men and 36 (15%) were women. More men received LVADs (70%) than women (61%) and more men were alive at 6 months compared to women, both among those who received LVADs (87% vs 82%) and those who did not (74% vs 50%) (p = 0.002). Compared to men, women were more likely to have decision regret, depressive symptoms and perceived stress at baseline but not at follow-up. At 6-month follow-up, men experience improvements in decisional conflict, acceptance of illness, struggle with illness, and perceived stress–none of those improvements were noted amongst women. Compared to men who received LVADs, women receiving LVADs had worse decision regret and depressive symptoms at baseline and worse acceptance of illness and perceived stress at six months. Men who received LVADs experienced improvement in decisional conflict and perceived stress, while women did not experience these improvements. Both men and women who received LVADs experienced improvement in depressive symptoms. Quality of life as assessed by EuroQol visual analog scale improved for both men and women who received LVADs but not for those who did not receive LVADs; no gender differences in quality of life were noted.

**Conclusion:**

Women require greater decisional support at time of decision to undergo LVAD implantation and subsequently might benefit from more intensive psychosocial support.

## Introduction

As left ventricular assist devices (LVADs) continue to improve, their utilization in women has remained low [1]. Women also experience higher rates of LVAD complications and death [2]. However, how gender impacts decision making regarding LVAD implantation and the effects of that decision remains unknown. In this study, we analyzed the Multicenter Trial of a Shared Decision Support Intervention for Patients and their Caregivers Offered Destination Therapy for End-Stage Heart Failure (DECIDE-LVAD) to assess the association of gender with differences in decisional outcomes among LVAD recipients and non-recipients [3].

The DECIDE-LVAD (NCT02344576) trial tested the effectiveness of a shared decision support intervention consisting of a pamphlet and video decision aid and clinician-directed support training. DECIDE-LVAD used a hospital-level, randomized phased rollout in six US LVAD implant sites [3]. Eligible patients were adults with advanced HF who were actively considering primary implantation of a destination therapy LVAD. Non-recipients included patients who chose not to get an LVAD or were deemed ineligible for LVAD.

Our primary outcomes of interest were decision regret and decisional conflict and secondary outcomes included depressive symptoms via Patient Health Questionnaire-2 (PHQ-2), PEACE Struggle with Illness subscale and PEACE Acceptance of Illness subscale, Perceived Stress, EuroQOL Visual Analog Scale and mortality [4, 5]. Enrolled patients were surveyed at baseline, immediately following evaluation, and at 1 and 6 months. We compared baseline characteristics and survey responses by self-identified gender and LVAD status using chi square test for categorical variables and Analysis of Variance (ANOVA) for continuous variables. To estimate outcome scores overtime by gender–random effect for center and fixed effect for stepwise study periods and 4 time-points (baseline 1, baseline 2, 1-months, and 6-months). We also adjusted for years since heart failure diagnosed and hemoglobin levels (p-value for baseline characteristics < 0.25). We fit the model on the basis of gender and then subset of LVAD recipients. Normality was assessed using Skewness and kurtosis tests with a null hypothesis that data follows equal distribution (p = 0.55)–failed to reject null hypothesis. All analysis were performed using Stata version 17.0 software (Stata Corporation, College Station, TX, USA).

Of 248 patients enrolled, 9 withdrew from all study assessments. Of the remaining 239 patients enrolled, 203 (84.9%) were men and 36 (15.1%) were women **(Table 1).** More men were implanted with an LVAD by 6 months (70%, n = 142) than women (61%, n = 22). More men were alive at 6 months compared to women, both among those who received LVADs (87% vs 82%) and those who did not (74% vs 50%) (p = 0.002).

**Table 1:** Characteristics of patients by gender at initiation of LVAD evaluation (Baseline) and by LVAD implantation status at 6 months.

		Men/LVAD (N = 142)	Men/No LVAD (N = 58)	Women/LVAD (N = 22)	Women/No LVAD (N = 13)	P value	Missing
**Age (years)**		63.4 (9.8)	63.6 (10.3)	62.3(8.4)	60.2 (9.0)	0.66	0
**Race**	*Missing*	0 (0.0%)	1 (1.7%)	0 (0.0%)	0 (0.0%)	0.71	1
	*NH-White*	118 (83.1%)	46 (79.3%)	17 (77.3%)	10 (76.9%)		
	*Black*	19 (13.4%)	6 (10.3%)	4 (18.2%)	2 (15.4%)		
	*Other*	5 (3.5%)	5 (8.6%)	1 (4.5%)	1 (7.1%)		
**Years since CHF**	*<2 years*	10 (7.0%)	12 (20.7%)	3 (13.6%)	2 (15.4%)	0.099	0
	*2–4 years*	125 (88.0%)	43 (74.1%)	18 (81.8%)	9 (69.2%)		
	*≥ 4 years*	7 (4.9%)	3 (5.2%)	1 (4.5%)	2 (15.4%)		
**Status at enrollment?**	*Outpatient*	34 (23.9%)	14 (24.1%)	3 (13.6%)	4 (30.8%)	0.70	0
	*Inpatient*	72 (50.7%)	32 (55.2%)	15 (68.2%)	5 (38.5%)		
	*ICU*	36 (25.4%)	12 (20.7%)	4 (18.2%)	4 (30.8%)		
**Sodium (mmol/L)**		135.9 (4.4)	134.9 (4.4)	135.9 (4.9)	136.2 (5.3)	0.63	5
**Creatinine (mg/dL)**		1.6 (.6)	1.6 (.6)	1.3 (.5)	1.4 (.5)	0.26	5
**Albumin (g/dL)**		3.5 (.6)	3.6 (.6)	3.5 (.5)	3.6 (.5)	0.68	26
**Hemoglobin (g/dL)**		12.0 (2.2)	12.6 (2.4)	11.4 (2.5)	11.5 (2.6)	0.17	6
**Patient status at 6 months**	*Alive*	124 (87.3%)	44 (75.9%)	18 (81.8%)	7 (53.8%)	0.01	0
	*Dead*	18 (12.7%)	14 (24.1%)	4 (18.2%)	6 (46.2%)		

Data are presented as mean (SD) for continuous measures, and n (%) for categorical measures. Abbreviations: ICU–intensive care unit, NH–non-Hispanic. P values: ANOVA for continuous variables, chi-square test for categorical variables

**Fig 1:**
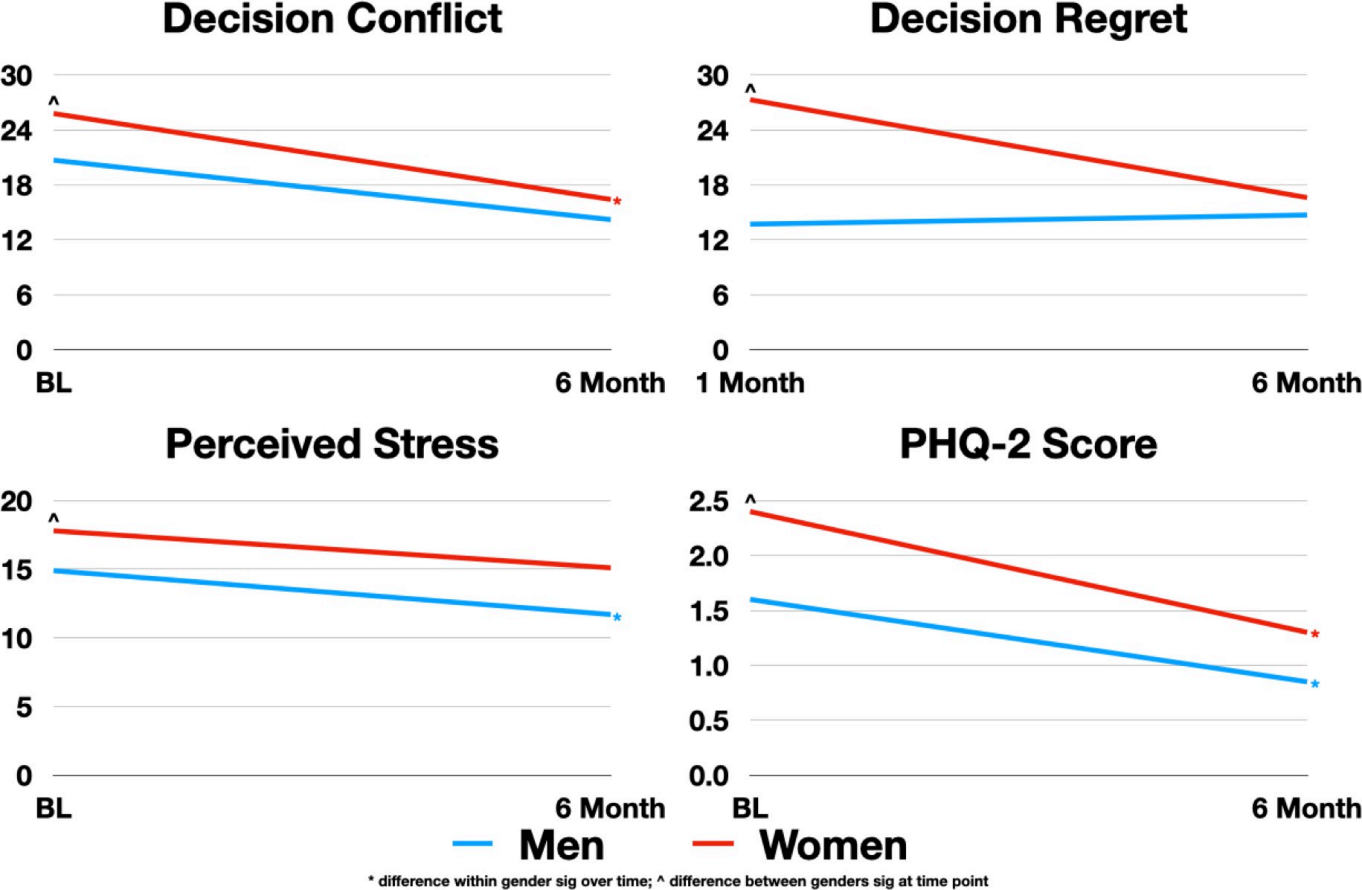
Differences in decision regret, decision conflict, perceived stress and depression between men and women considering LVAD therapy. Depression assessed using the Patient Health Questionnaire– 2 Scale. Lower scores better for all four measures. P value <0.05 considered statistically significant.

Overall compared to men, women were more likely to have decision regret, depressive symptoms and perceived stress at baseline though these differences were not noted at follow-up **(Fig 1 and Table 2)**. At 6-month follow-up, men saw improvements over baseline in decisional conflict, acceptance of illness, struggle with illness, and perceived stress, though none of those improvements were noted amongst women. Both men and women experienced improvement in depressive symptoms and quality of life (QoL) at 6 months. Compared to men who received an LVAD, women who received an LVAD had worse decision regret and depressive symptoms at baseline and worse acceptance of illness and perceived stress at six months. Men who received an LVAD experienced improvement after 6 months in decisional conflict and perceived stress, while women who received LVADs did not experience these improvements. Both men and women who received LVADs experienced improvement in depressive symptoms and QoL; however, men and women who did not receive an LVAD did not experience an improvement in QoL. Further, there were no gender differences in QoL between men and women in either group.

**Table 2:** Differences in adjusted decision outcomes at baseline and 6 months by gender and LVAD status.

Measure	Visit	Men (n = 200)	Women (n = 35)	P value	Men/ LVAD (n = 142)	Women/ LVAD (n = 22)	P value	Men/ No LVAD (n = 58)	Women/ No LVAD (n = 13)	P value
**Decisional Conflict (0–100)** *	*Baseline*	15.1 (8.7)	NA	0.059	2.68 (10.8)	NA	0.12	32.2 (12.7)	NA	0.17
	*6M*	11.6 (9.0)	NA	0.49	16.2 (9.5)	NA	0.16	3.6 (6.6)	NA	0.17
	*P value*	< 0.01	0.069		0.012	0.14		0.029	0.21	
**Decision Regret (0–100)** *	*1M*	25.1 (10.3)	NA		21.8 (10.6)	NA	0.04	43.0 (24.1)	NA	NA
	*6M*	NA	NA	0.28	NA	14.8 (9.5)	0.18	NA	NA	0.51
	*P value*	0.75	0.08		0.63	0.61		0.90	NA	
**PEACE Acceptance of Illness (5–20)** ^	*Baseline*	17.2 (1.3)	19.1 (3.2)	0.25	20.2 (1.6)	14.9 (4.0)	0.09	13.3 (2.23)	27.3 (5.0)	0.9
	*6M*	19.3 (1.5)	14.7 (4.0)	0.04	19.1 (1.7)	16.2 (3.7)	0.005	20.0 (0.8)	NA	0.45
	*P value*	0.053	0.94		0.21	0.71		0.074	0.40	
**PEACE Struggle With Illness (7–28)** *	*Baseline*	16.5 (2.1)	13.6 (4.7)	0.35	13.3 (2.8)	12.8 (6.5)	0.12	21.0 (2.87)	8.2 (6.2)	0.75
	*6M*	12.7 (2.8)	17.9 (8.1)	0.07	10.8 (3.1)	19.3 (4.8)	0.12	9.0 (2.14)	NA	0.048
	*P value*	0.008	0.86		0.009	0.26		0.63	0.25	
**Patient Health Questionnaire 2 (0–6)** *	*Baseline*	2.3 (0.84)	3.0 (2.1)	0.017	1.94 (1.1)	2.60 (2.5)	0.01	3.1 (1.3)	0.25 (2.94)	0.28
	*6M*	1.0 (0.92)	0.52 (1.7)	0.10	1.3 (1.1)	1.1 (1.5)	0.053	0.3 (0.5)	NA	0.92
	*P value*	<0.01	0.02		<0.01	0.013		0.24	0.09	
**Perceived Stress (0–40)** *	*Baseline*	19.9 (3.3)	21.9 (7.2)	0.043	18.9 (4.1)	29.3 (8.2)	0.06	20.4 (5.2)	8.1 (8.0)	0.1
	*6M*	10.8 (4.3)	12.1 (9.6)	0.02	8.3 (4.7)	11.0 (9.1)	<0.01	8.7 (3.2)	NA	0.20
	*P value*	<0.01	0.21		<0.01	0.22		0.96	0.10	
**EQ VAS (0–100)** ^	*Baseline*	48.0 (3.5)	39.5 (7.5)	0.56	46.8 (4.0)	35.6 (6.2)	0.48	52.3 (7.2)	55.0 (18.7)	0.59
	*6M*	68.2 (3.5)	60.5 (8.0)	0.68	69.1 (3.6)	59.8 (10.7)	0.32	59.3 (10.5)	86.7 (6.3)	0.38
	*P value*	< 0.01	< 0.01		< 0.01	< 0.01		0.09	0.17	

Values are adjusted mean (standard error) from linear mixed models. Mixed linear models analyzed using: Using fixed effects–stepwise study periods and 4 time-points (baseline 1, baseline 2, 1-months, 6-months) Random effects–study site. *Higher score is worse.

^Lower score is worse.

Compared to men, women with advanced HF considering LVADs are more likely to suffer from decisional regret, depressive symptoms, perceived stress and death. And while men experienced improvement in decisional conflict, acceptance of illness, struggle with illness and perceived stress, these improvements were not noted amongst women. While the number of women enrolled was small, several findings reached statistical significance. This study provides novel insight into the decision-making window for LVADs in women upstream of implantation that shows their unique struggles with the decision in comparison to men. The lack of differences in QoL as measured using EuroQol Visual Analog Scale suggest that these differences were not mediated by differences in somatic QoL, though our study could have benefited from further measurement of QoL using disease-specific measures such as the Kansas City Cardiomyopathy Questionnaire. Additionally, most studies of LVADs focus only on those who receive implantation, but we found in this cohort of all patients considered for LVAD that gender differences also carried over into the non-implanted group. This study highlights the need for support mechanisms for women considering LVAD in order to improve their decision-making and lived experience with an LVAD.
